# Multi-Resonant Metamaterial Absorber for Electromagnetic Absorption in S-, C-, X-, and Ku- Bands

**DOI:** 10.3390/s26103113

**Published:** 2026-05-14

**Authors:** Iftikhar Ud Din, Daud Khan, Sarosh Ahmad, Tayeb A. Denidni

**Affiliations:** National Institute for Scientific Research (INRS), University du Quebec, Montreal, QC H5A 1K6, Canada; daud.khan@inrs.ca (D.K.); sarosh.ahmad@inrs.ca (S.A.); tayeb.denidni@inrs.ca (T.A.D.)

**Keywords:** metamaterial, absorber, resonator, hybrid structure, surface impedance, free space

## Abstract

This work introduces a compact multi-resonant metamaterial absorber designed to achieve efficient electromagnetic absorption over several microwave frequency bands. The proposed configuration is based on a hybrid resonator arrangement that promotes strong electromagnetic interaction and enables multiple resonant modes within a single unit cell. Consequently, six distinct absorption peaks are obtained at 2.4, 5.21, 6.88, 9.77, 12.61, and 14.99 GHz, covering S-, C-, X-, and Ku-band applications. The absorber exhibits high absorption performance, exceeding 97% across most operating frequencies and slightly lower value is observed of 91.13% at 12.61 GHz, which indicates effective impedance matching with free space and efficient energy dissipation mechanisms. The absorption characteristics are further examined through surface current distributions, electric field confinement, and effective medium analysis, demonstrating that the multi-band response originates from the interaction of multiple resonant elements and intrinsic material losses. Moreover, the proposed structure maintains stable performance for different polarization angles and oblique wave incidence, confirming its polarization-insensitive and angularly stable behavior. To validate the design, a prototype is fabricated and experimentally characterized using a free-space measurement setup, showing close agreement with the simulated results. The compact geometry, low fabrication cost, and scalability of the proposed absorber make it a promising candidate for applications such as electromagnetic interference mitigation, radar cross-section reduction, and modern wireless communication systems.

## 1. Introduction

The rapid growth of wireless communication systems, radar technologies, and compact electronic devices has resulted in a substantial increase in electromagnetic (EM) radiation across the microwave frequency spectrum [1,2]. Contemporary technologies such as Wi-Fi, sub-6 GHz and millimeter-wave (mmWave) 5G networks, satellite communication systems, Internet of Things (IoT) devices, and advanced sensing platforms operate concurrently over multiple frequency bands. This spectral congestion leads to severe electromagnetic as well as radio-frequency interference effects, signal distortion, and overall system performance degradation [3,4]. Consequently, the development of efficient electromagnetic absorbers capable of suppressing unwanted reflections and mitigating interference has become a critical research challenge [5]. Conventional microwave absorbers, including Salisbury screens, Jaumann absorbers, and Dallenbach layers, typically employ resistive sheets combined with quarter-wavelength spacing to achieve absorption at specific frequencies [6,7]. While these structures provide reasonable absorption performance, they inherently suffer from large physical thickness, narrow operating bandwidth, and limited tunability, particularly at lower microwave frequencies [8]. Such drawbacks significantly restrict their integration into modern compact, lightweight, and multifunctional electronic systems required for advanced wireless and stealth applications [9]. Many metamaterial absorbers (MMAs) have emerged as promising alternatives to conventional absorbers as a result of their ability to manipulate electromagnetic waves using artificially engineered subwavelength resonant elements rather than relying solely on bulk material properties [10,11]. Since the demonstration of a close-to-unity metamaterial absorber utilizing on impedance-matching principles by Landy et al., MMAs have attracted considerable research interest across microwave, terahertz, infrared, and optical frequency regimes [12,13]. By simultaneously tailoring the effective electric permittivity and magnetic permeability, MMAs can significantly suppress both reflection and transmission, thereby enabling near-unity absorption within electrically thin and compact structures [14]. Typically, metamaterial absorbers are realized through a three-layer arrangement consisting of a structured metallic surface, a dielectric spacer, and a conductive ground layer [15]. The ground plane effectively blocks electromagnetic transmission, while the interaction between the resonant top layer and the dielectric substrate facilitates impedance matching with free space. As a result, incident electromagnetic energy is efficiently dissipated through dielectric and ohmic losses [16,17]. By tuning the resonator geometry, substrate characteristics, and electromagnetic coupling mechanisms, MMAs are capable of operating over desired frequencies with high absorption efficiency [18]. Based on their spectral response, metamaterial absorbers are generally classified into single-band, multiband, and broadband categories [19]. Single-band absorbers offer high frequency selectivity and are widely employed in sensing and narrowband EMI suppression applications [20]. However, the growing demand for multifunctional and multi-standard wireless systems has shifted research interest toward multiband and broadband absorbers capable of operating over multiple discrete or continuous frequency ranges [21]. Multiband metamaterial absorbers provide an effective solution for addressing several wireless standards simultaneously without increasing structural complexity or device footprint [22]. Numerous multiband metamaterial absorbers have been reported using nested resonators, hybrid geometries, and coupled resonant elements [23,24]. Recent studies have also investigated broadband metamaterial absorbers based on hybrid metallic and graphene metasurfaces. For instance, the work reported in [25] employed metallic and graphene resonators separated by SiO^2^ dielectric layers to achieve broadband absorption through combined surface plasmon, cavity, and gap resonance effects. The proposed structure achieved absorptance above 80% over the 0.95–1.95 THz range, demonstrating the effectiveness of hybrid resonant mechanisms for advanced electromagnetic absorption applications. The authors have demonstrated the growing potential of metamaterial absorbers in sensing, energy harvesting, and acoustic applications. A multi-resonant metamaterial absorber sensor reported in [26] achieved broadband gas detection over the 2–12 GHz range with four resonances at 2.95, 4.95, 7.05, and 11.48 GHz, exhibiting high sensitivity and ppm-level gas detection capability. Broadband metamaterial absorbers have also been explored for improving optical absorption and efficiency in organic solar cells, where polarization-insensitive and wide-angle absorption characteristics were achieved using various BMMA configurations [27]. In addition, acoustic metamaterials based on hybrid honeycomb–Helmholtz resonator structures have demonstrated broadband low-frequency sound absorption through the coupling of multiple resonant modes [28]. These studies highlight the versatility of metamaterial absorbers across electromagnetic and acoustic domains and motivate the development of compact, high-efficiency multiband absorbers for microwave applications. Dual- and triple-band absorbers targeting Wi-Fi, sub-6 GHz 5G, and X-band radar applications have demonstrated absorption levels exceeding 90% at selected frequencies [29,30]. Nevertheless, many existing designs remain limited by a small number of resonant bands, reduced absorption efficiency at higher frequencies, or inadequate scalability across widely separated microwave bands [31]. Although recent studies have explored absorbers operating in the Ku-band and higher microwave frequencies for radar stealth, satellite communications, and EMI shielding [32,33], achieving stable multiband absorption that simultaneously covers lower microwave bands bands centered around 2.4 GHz and spanning 5–7 GHz remains challenging due to increased electrical size and impedance mismatch issues [34]. Therefore, compact multiband metamaterial absorbers capable of delivering high absorption efficiency across both lower and upper microwave bands are still highly desirable [35].

This study presents a compact and efficient microwave metamaterial absorber with a multi-resonant configuration exhibiting six well-defined absorption bands at 2.4 GHz, 5.21 GHz, 6.88 GHz, 9.77 GHz, 12.61 GHz, and 14.99 GHz, covering the S-, C-, X-, and Ku-frequency ranges. At these resonances, the absorber achieves absorption efficiencies exceeding 97%, with peak absorptivity values of approximately 98.23%, 97.98%, 98.23%, 97.73%, 91.13%, and 97.43%, respectively. Compared with previously reported multiband absorbers, the proposed design provides wider spectral coverage and improved absorption performance [36]. The novelty of the proposed work lies in the realization of hexaband operation, compact size, polarization-insensitive behavior, angular stability, and scalable multi-resonant performance within a single-layer planar structure. Unlike conventional dual-, triple-, or five-band absorbers, the proposed absorber employs a hybrid concentric arrangement of square, circular, dodecagonal, and diamond-shaped resonators to generate multiple resonant modes through controlled electromagnetic coupling. The optimized unit cell maintains a compact size of only $13\times 13\;{\mathrm{mm}}^{2}$, making it suitable for integration into modern wireless and stealth platforms. The observed multiband absorption behavior originates from the excitation of multiple resonant modes that provide improved impedance matching with free space at each operating frequency [37,38]. This mechanism suppresses reflection and minimizes transmission, resulting in efficient electromagnetic energy dissipation [39]. The significance of the proposed six-band response over conventional 3–5 band absorbers is that it simultaneously supports applications including Wi-Fi, sub-6 GHz communications, radar systems, EMI suppression, and Ku-band operations within a single metasurface. This broader spectral coverage reduces the need for multiple independent absorber structures while preserving low structural complexity and a thin profile. Furthermore, the proposed design strategy is scalable, since the resonance frequencies can be systematically tuned through geometric scaling and LC-coupling control for other microwave and millimeter-wave applications [40,41].

The remainder of this paper is organized as follows. Section 2 presents the electromagnetic excitation mechanism and the optimization of the proposed layered metamaterial absorber architecture. Section 3 details the design configuration, numerical modeling, and optimization procedure used to achieve the desired absorption performance. Section 4 explains the fundamental absorption mechanism responsible for the observed resonant behavior. Section 5 describe equivalent circuit modeling and resonance mechanism and Section 6 investigates the polarization-independent absorption response of the proposed structure, while Section 7 analyzes its angular stability under oblique incidence for different polarization modes. Section 8 examines the distributions of surface currents and electric fields to provide physical insight into the resonance formation and energy dissipation mechanisms. Section 9 discusses the frequency-dependent effective electromagnetic parameters of the absorber, including permittivity, permeability, and normalized impedance. Section 10 presents the experimental validation through measured absorption results, followed by Section 11, which provides a comparative analysis with previously reported designs. Finally, Section 12 concludes the paper by summarizing the key findings and main contributions of the proposed metamaterial absorber.

## 2. Electromagnetic Excitation Mechanism and Optimization of the Layered Architecture

**Figure 1:**
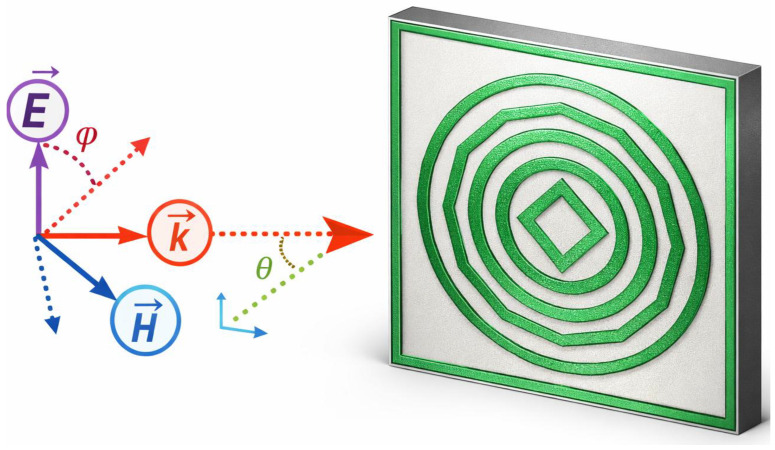
Illustration how an incident electromagnetic wave interacts with the proposed multilayer absorber, highlighting the orientations of the electric field, magnetic field, and propagation direction relative to the structure.

Figure 1 illustrates the electromagnetic configuration and Absorption Profile of the multiband Artificial EM absorber operating in the microwave frequency spectrum. The structure exhibits multiple distinct absorption resonances within the GHz spectrum, with pronounced absorption peaks occurring at 2.4 GHz, 5.21 GHz, 6.88 GHz, 9.77 GHz, 12.61 GHz, and 14.99 GHz, respectively. At these frequencies, the absorber achieves high absorption efficiency exceeding 97%, with peak absorptivity values of approximately 98.23%, 97.98%, 98.23%, 97.73%, 91.13%, and 97.43%, respectively. These results confirm that the proposed metasurface supports multiple distinct resonant modes, and each of them is responsible for efficient electromagnetic energy dissipation at its corresponding resonance frequency. Unlike terahertz absorbers that typically require optical or spectroscopic characterization techniques, the proposed design operates in the microwave domain. As a result, its experimental validation can be conveniently performed using vector network analyzer (VNA)-based free-space measurement setups. The absorber is composed of a metasurface unit cell formed by a patterned metallic resonator developed on an FR-4 dielectric layer. The resonator geometry follows a concentric hybrid configuration that combines square, circular, dodecagonal, and diamond-shaped elements. Intense electromagnetic interaction among the resonators gives rise to multiple resonances, contributing to stable performance multiband absorption across the 1–16 GHz frequency range. At each operating band, the metasurface achieves effective impedance matching with free space, which significantly suppresses reflection while maintaining minimized transmission because of the metallic ground layer. Consequently, near-perfect absorption is obtained at distinct resonance frequency points. As depicted in Figure 1, The absorber response under incident electromagnetic-wave excitation represented by the electric field (*E*), magnetic field (*H*), and propagation vector (*k*), which are defined according to the selected incidence angle (θ) and polarization angle (ϕ). These excitation conditions are essential for evaluating the angular stability, polarization insensitivity, and resonance robustness of the absorber under realistic operating scenarios. Furthermore, strong localization of the electromagnetic fields within the metasurface at resonance enhances both dielectric and conductive losses within the lossy substrate, directly contributing to the observed high absorption levels. The ability of the proposed design to maintain consistently high absorptivity across multiple discrete microwave bands highlights its effectiveness and confirms its suitability for real-world applications including electromagnetic interference mitigation, a reduction in the radar cross-section, and microwave stealth technologies.

## 3. Design Configuration, Simulation and Optimization

**Figure 2:**
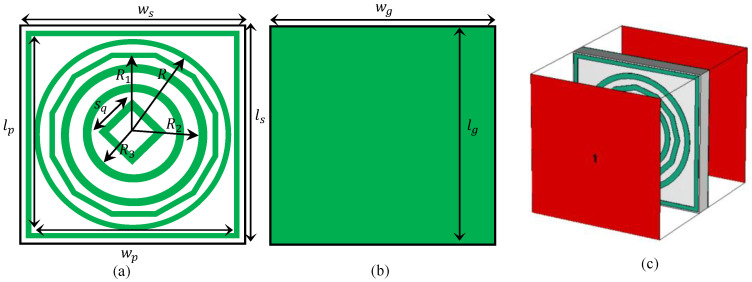
Design configuration of the proposed MMA absorber.

The unit cell configuration of the developed metamaterial absorber is presented in Figure 2, where the front and backside layouts are shown in Figure 2a and Figure 2b, respectively. The structure is composed of three distinct layers, including a patterned conductive layer on the top, a dielectric medium in the middle, and a solid metallic layer at the bottom. The bottom metallic sheet is implemented using copper and acts as a reflector, preventing the transmission of incident electromagnetic waves. This ensures that the transmission coefficient remains negligible, which is a fundamental requirement for maximizing absorption performance. The top layer consists of a copper-based resonating pattern with a conductivity of $5.8\times 10^{7}\;S/m$ and a thickness of 0.035 mm. This layer is responsible for inducing strong localized currents, which contribute to resonant energy confinement with reduced resistive losses. An FR-4 substrate is used as the intermediate layer, characterized by a relative permittivity of $\varepsilon_{r}=4.4$ and a thickness of 1.6 mm. This dielectric spacing plays an essential role in regulating the interaction between the resonator and the ground plane, enabling proper impedance matching with free space. The structural dimensions were adjusted through a detailed parametric analysis to generate multiple resonant modes associated with both electric and magnetic responses. This design strategy leads to the realization of multi-band absorption behavior. All geometric parameters listed in Table 1 have been clearly defined and labeled in the corresponding geometry illustration shown in Figure 2, while $w_{p}$ and $l_{p}$ denote the width and length of the outer resonator, respectively. In addition, sq corresponds to the side length of the central diamond-shaped resonator element. Furthermore, the parameters *R*, $R_{1}$, $R_{2}$, and $R_{3}$ represent the radii of the concentric resonator rings, whereas $w_{s}$, $l_{s}$, $w_{g}$, and $l_{g}$ correspond to the substrate and ground-plane dimensions. All structural parameters have now been explicitly labeled in Figure 2. The final optimized values of the design variables are provided in Table 1. This design strategy leads to the realization of multi-band absorption behavior. The final optimized values of the design variables are provided in Table 1. Compared with previously reported absorbers in Refs. [38,39,40,41,42,43,44,45], the novelty of the proposed absorber lies in the integration of four distinct resonator geometries, namely the square frame, concentric circular rings, dodecagonal ring, and central diamond resonator, within a single compact unit cell. This hybrid resonator configuration enhances electromagnetic coupling and enables the excitation of six distinct resonant modes, resulting in hexaband absorption at 2.4, 5.21, 6.88, 9.77, 12.61, and 14.99 GHz. In contrast to previous studies, which mainly reported dual-, triple-, or five-band responses, the proposed design simultaneously covers the S-, C-, X-, and Ku-bands while maintaining absorptivity above 97% at most resonance frequencies. Furthermore, the absorber demonstrates polarization-insensitive behavior and stable angular performance up to $45^{{}^{\circ}}$ incidence for both TE and TM polarizations, making it more suitable for practical microwave and radar applications.

Figure 2c shows that Full-wave electromagnetic simulations were performed by modeling a one-unit cell under periodic conditions applied at the boundaries in the transverse (*x*–*y*) directions, thereby emulating an infinite two-dimensional metasurface array. Open (radiation) boundary conditions were introduced along the *z*-direction to accurately represent open-space wave propagation. The absorber was excited using a normally incident plane wave over the frequency range of 1–16 GHz, corresponding to the intended operational frequency range of the MMA. All numerical analyses were executed using the frequency-domain solver available in CST Microwave Studio to ensure accurate characterization of the steady-state electromagnetic response of the absorber. Floquet ports were employed to excite the structure, preserving the periodic nature of the incident wavefront and enabling precise extraction of the scattering parameters, while also allowing for the analysis of oblique incidence. To ensure numerical accuracy, a finite-element-based discretizations scheme was adopted, with adaptive mesh refinement applied in regions exhibiting strong electromagnetic field variations. This meshing strategy provides an optimal trade-off between computational efficiency and accuracy. The frequency-dependent scattering parameters obtained from the simulations were subsequently used to evaluate the absorption coefficient of the proposed structure. The absorption performance of the presented metamaterial structure is evaluated using the well-known expression

(1) $$A=1-|S_{11}{|}^{2}-{\left|S_{21}\right|}^{2}$$

In this expression, $|S_{11}{|}^{2}$ corresponds to the reflected power ratio, while $|S_{21}{|}^{2}$ denotes the transmitted power ratio through the structure. For the proposed absorber, a continuous metallic ground layer is incorporated at the bottom, which effectively blocks wave transmission. Consequently, the transmission term becomes negligible, i.e., $|S_{21}{|}^{2}\approx 0$. Under this condition, the absorption expression can be reduced to

(2) $$A=1-|S_{11}{|}^{2}$$

indicating that the absorption performance is controlled by reflection minimization. The proposed structure exhibits multiple resonant absorption modes across the microwave frequency spectrum. Distinct absorption peaks are observed at 2.4 GHz, 5.21 GHz, 6.88 GHz, 9.77 GHz, 12.61 GHz, and 14.99 GHz. At these frequencies, the absorber demonstrates strong absorption performance exceeding 97%, with peak absorptivity values of approximately 98.23%, 97.98%, 98.23%, 97.73%, 91.13%, and 97.43%, respectively. These results confirm that the metasurface supports multiple electromagnetic resonances originating from different current paths and LC coupling mechanisms within the unit cell.

**Figure 3:**
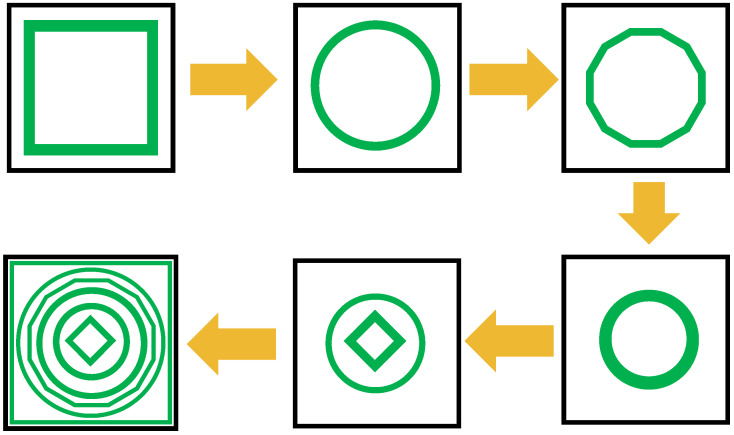
Design steps of the presented Absorber.

**Figure 4:**
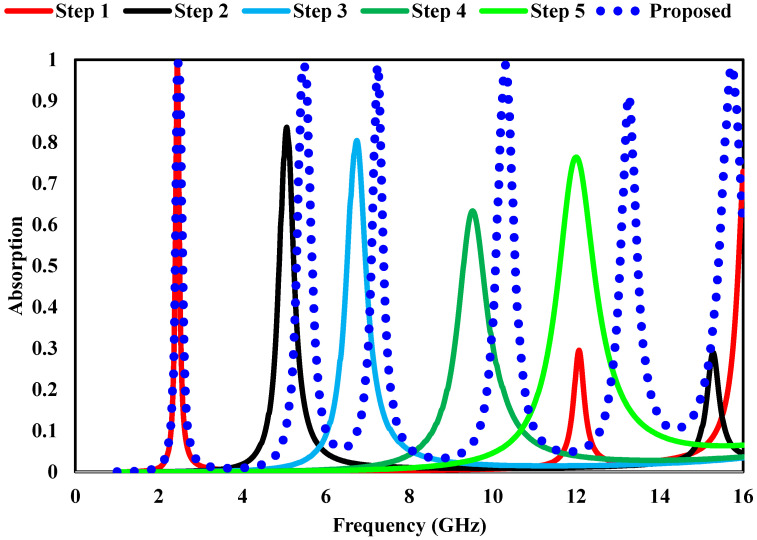
Simulated response of the proposed optimization steps.

Figure 3 illustrates the progressive optimization of the Geometric layout of the unit cell, while the corresponding absorption response at each design stage is presented to elucidate the contribution of individual resonant features. In Step 1, the initial configuration exhibits a strong absorption resonance at 2.4 GHz with a near-unity absorptivity of 0.99, indicating effective impedance matching in the lower microwave band. With the introduction of additional structural elements in Step 2, a new resonance appears at 4.9 GHz, achieving an absorption level of approximately 0.80. Further geometric refinement in Step 3 shifts the resonance to 6.49 GHz, where a comparable absorptivity of 0.80 is maintained, demonstrating stable mid-band absorption behavior. In Step 4, the structure supports maximum absorption observed at 9 GHz with a moderate absorptivity of 0.60. Subsequently, Step 5 introduces an additional resonance at 12 GHz with an improved absorption level of 0.76. These intermediate stages clearly illustrate how successive geometric modifications activate new resonant modes and progressively expand the operational bandwidth. Based on this systematic design evolution, the final optimized layout exhibits multiple well-defined absorption resonances across the microwave spectrum, as depicted in Figure 4. This multi-resonant absorption response confirms that the final configuration effectively integrates the individual resonant contributions identified during the design stages, resulting in enhanced absorption strength and wide spectral coverage suitable for practical microwave applications.

## 4. Absorption Mechanism

**Figure 5:**
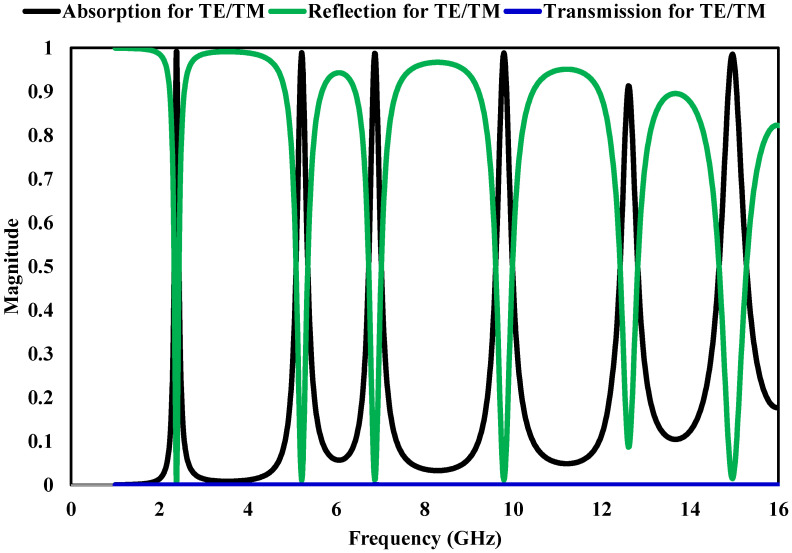
Absorption spectra evaluated for electromagnetic waves with both TE and TM polarization states when incident normally on the structure.

Figure 5 presents the simulated scattering parameters together with the corresponding absorption behavior of the proposed unit cell. The results include the response under both transverse electric (TE) and transverse magnetic (TM) excitations. The absorption profile, derived from the S-parameters, indicates the presence of several distinct resonant peaks distributed over the microwave frequency spectrum. Significant absorption levels are achieved at approximately 2.4 GHz, 5.21 GHz, 6.88 GHz, 9.77 GHz, 12.61 GHz, and 14.99 GHz, where the absorptivity remains above 97% for most of these resonances. At these frequencies, the structure approaches near-complete absorption, which suggests effective impedance matching between the metasurface and free space.To further explain the physical origin of the resonant absorption peaks observed during the design evolution shown in Figure 3, we perform analytical relations based on equivalent LC resonance theory. The resonant frequency of each metallic resonator is primarily governed by its effective inductance (L), associated with the surface current path length, and capacitance (C), generated by the electromagnetic coupling gaps between adjacent resonators. Accordingly, the resonance frequency can be estimated as

(3) $$f_{r}\approx \frac{1}{2\pi \sqrt{LC}}$$

For ring- and loop-type resonators, the inductance is approximately proportional to the resonator perimeter, whereas the capacitance depends on the coupling gap spacing and the effective dielectric environment. Consequently, the resonance frequency using

(4) $$f_{r}\propto \frac{1}{L_{\mathrm{eff}}\sqrt{\varepsilon_{\mathrm{eff}}}}$$

where $L_{\mathrm{eff}}$ represents the effective current path length and $\varepsilon_{\mathrm{eff}}$ denotes the effective dielectric constant. Based on this relationship, the larger outer square and circular resonators produce lower-frequency resonances at around 2.4 GHz and 5.21 GHz due to their longer current paths, whereas the progressively smaller inner rings and the central diamond resonator generate the higher-frequency resonances at 9.77, 12.61, and 14.99 GHz. In addition metamaterial absorbers, such behavior is typically obtained when both reflection and transmission are minimized. Furthermore, the responses obtained under TE and TM polarizations closely overlap, demonstrating that the design maintains polarization-insensitive performance. The combination of copper layers and the FR-4 dielectric substrate contributes to consistent absorption efficiency across the operating bands. Overall, the unit cell exhibits multiple resonant absorption peaks over a wide frequency range, indicating its suitability for broadband microwave applications where stable and polarization-independent absorption is required.

## 5. Equivalent Circuit Modeling and Resonance Mechanism

**Figure 6:**
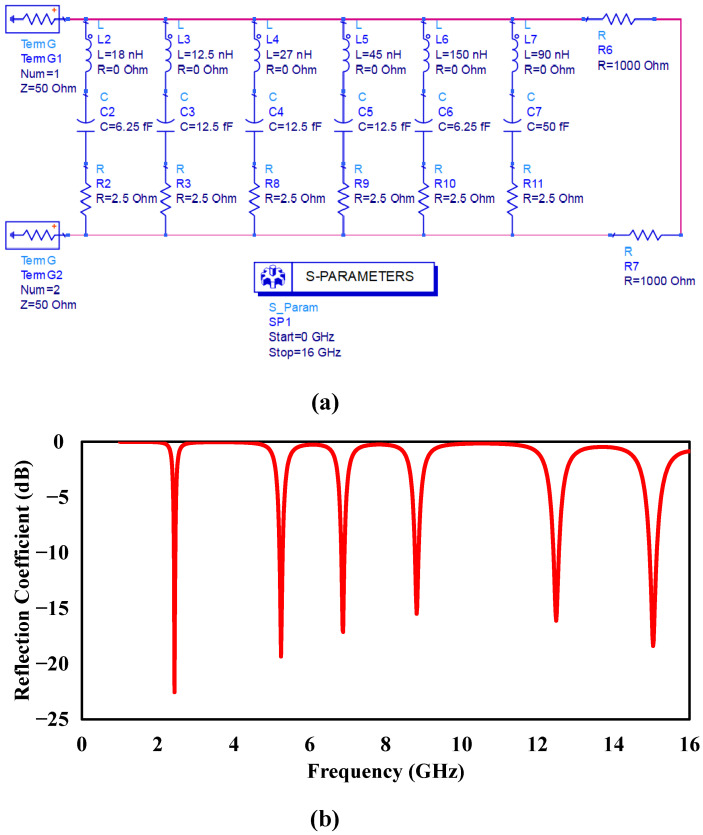
(**a**) Equivalent LC circuit model of the proposed multi-resonant metamaterial absorber and (**b**) corresponding reflection coefficient response showing six resonance modes.

To provide a more rigorous interpretation of the absorption behavior, the proposed metamaterial absorber is analyzed using an equivalent LC circuit model together with impedance-matching theory, as illustrated in Figure 6. The equivalent circuit consists of multiple parallel RLC resonant branches connected along the transmission path, where each branch corresponds to a distinct resonant mode generated by the concentric resonator elements of the absorber. In this model, the inductive components represent the effective surface-current paths associated with the metallic resonators, while the capacitive components originate from the coupling gaps between adjacent resonators and the dielectric substrate. Consequently, larger outer resonators generate lower-frequency resonances due to their longer current paths, whereas the smaller inner resonators support higher-frequency resonant modes. The reflection-coefficient response obtained from the equivalent circuit model is presented in Figure 6b, where multiple resonance dips are observed across the operating frequency range. These resonances closely correspond to the simulated absorption peaks of the proposed absorber, confirming the validity of the developed circuit representation.

## 6. Polarization-Independent Absorption Response

**Figure 7:**
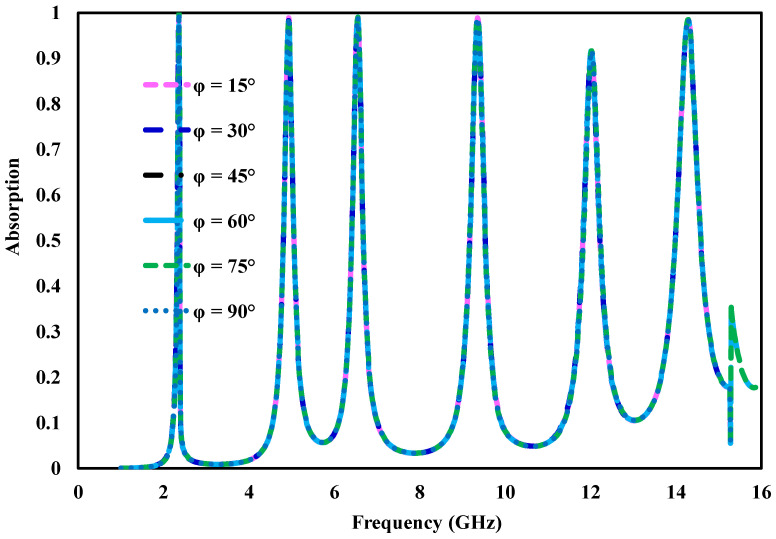
Simulated absorptivity response of the proposed metamaterial absorber for different polarization angles.

Polarization independence and angular robustness are critical indicators when evaluating the performance of electromagnetic absorbers. A well-designed absorber exhibits consistent absorption regardless of the orientation of the incident electric field. Figure 7 illustrates the simulated absorption response of the proposed structure for various polarization angles ($\phi =15^{{}^{\circ}},30^{{}^{\circ}},45^{{}^{\circ}},60^{{}^{\circ}},75^{{}^{\circ}},$ and $90^{{}^{\circ}}$) under normal incidence. The obtained results show that the absorption peaks remain nearly unaffected across all polarization states, demonstrating that the structure operates independently of polarization. This behavior is attributed to the symmetric configuration of the unit cell. Structures with rotational symmetry are known to provide identical electromagnetic responses for different polarization orientations. In the present design, the fourfold symmetry ensures uniform interaction with the incident wave along both orthogonal directions. As a result, the absorber preserves stable absorption characteristics over a wide range of polarization angles.

## 7. Angular-Dependent Absorption Characteristics

**Figure 8:**
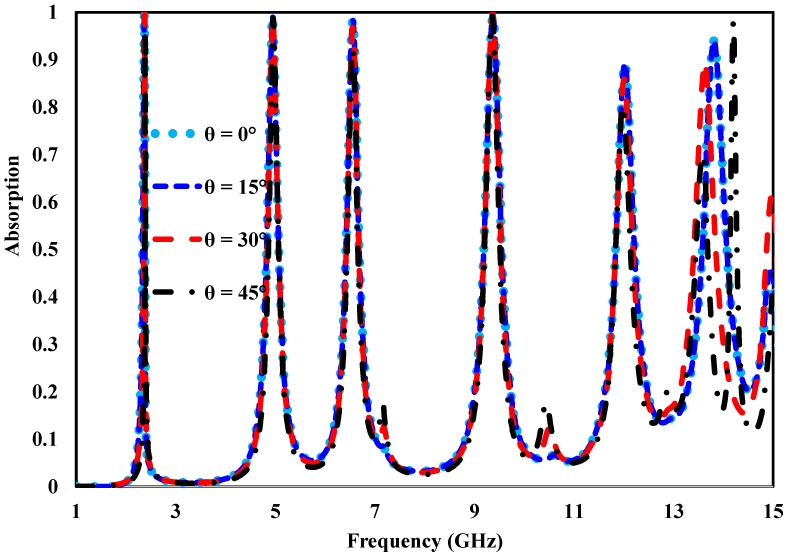
Simulated absorptivity response of the proposed metamaterial absorber under different incidence angles (θ).

In practical electromagnetic environments, incident waves may arrive from different directions rather than only normal incidence. To examine this behavior, the response of the proposed multi-band absorber was investigated under oblique incidence conditions for both transverse electric (TE) and transverse magnetic (TM) polarizations. Figure 8 presents the simulated absorption results for incidence angles of $\theta =0^{{}^{\circ}},15^{{}^{\circ}},30^{{}^{\circ}},$ and $45^{{}^{\circ}}$ for both polarization modes. It can be observed that the absorber maintains strong absorption performance, with values remaining above 85% across the considered angular range. Such behavior indicates a high level of angular stability, which is an important requirement for practical absorber applications. As the incident angle increases, a slight reduction in absorption efficiency is noticeable. In particular, minor variations in the absorption spectrum appear at higher frequencies. This effect is mainly attributed to changes in the distribution of the electromagnetic fields and the resulting coupling between resonant elements, which becomes more pronounced at oblique incidence angles, where variations in field components lead to impedance mismatch at larger angles. Despite these variations, the absorber continues to demonstrate reliable performance over a wide range of incidence angles for both TE and TM polarizations. This confirms its suitability for real-world microwave and radar systems, where the direction of incoming waves cannot be controlled.

## 8. Surface Currents and Electric Field Distributions

**Figure 9:**
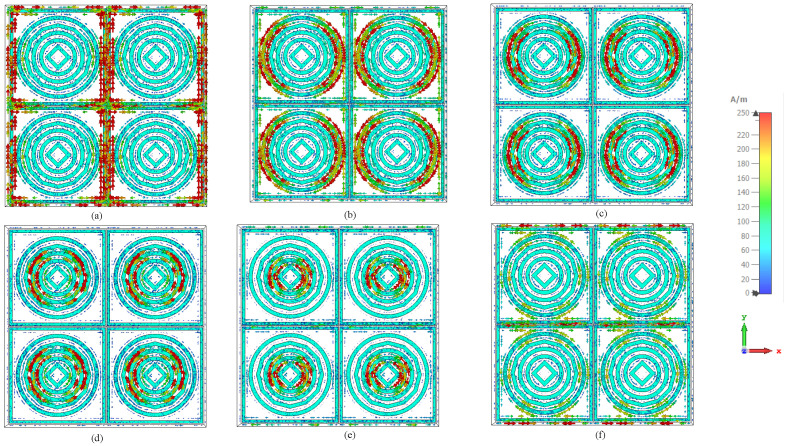
(**a**–**f**) Surface current responses of the proposed multi band absorber at 2.38, 5.2, 6.8, 9.7, 12.6, and 14.96 GHz, showing the evolution of current concentration across the outer, intermediate, and inner resonant structures.

**Figure 10:**
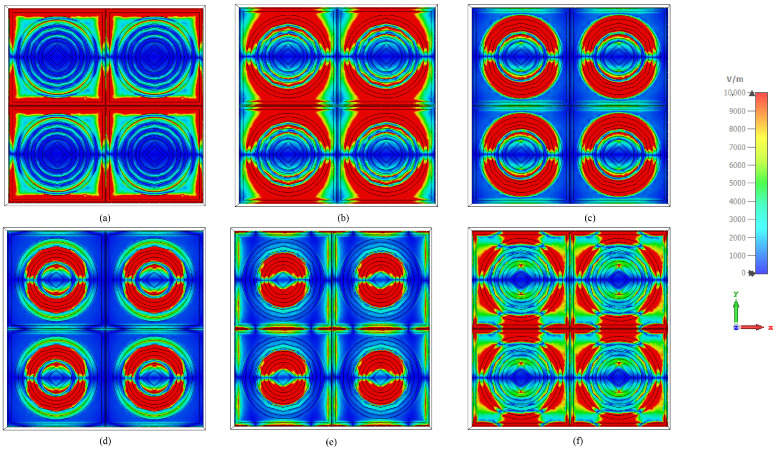
(**a**–**f**) Electric-field responses of the proposed hexaband absorber at 2.38, 5.2, 6.8, 9.7, 12.6, and 14.96 GHz, showing the evolution of field concentration across the outer, intermediate, and inner resonant structures.

Figure 9a–f illustrate the surface current distributions of the proposed absorber at 2.38, 5.2, 6.8, 9.7, 12.6, and 14.96 GHz, respectively, where the arrow density and color intensity represent the magnitude and direction of the induced currents. At 2.38 GHz, strong currents are predominantly confined along the outer square resonator and inter-cell metallic boundaries, forming large loop-like current paths. This indicates that the lowest-frequency resonance is mainly governed by the outer resonator and inter-element electromagnetic coupling. As the frequency increases to 5.2 GHz, the dominant excitation shifts toward the outer circular ring, where symmetric circulating currents are observed around the ring perimeter, confirming the excitation of a resonant loop-current mode. At 6.8 GHz, the current distribution becomes strongly localized on the middle circular ring, demonstrating that this resonator primarily contributes to the corresponding absorption band. At 9.7 GHz, the current concentration further contracts toward the inner circular ring, where compact circulating currents indicate the excitation of a higher-order localized resonant mode. At 12.6 GHz, intense currents are mainly concentrated around the central diamond-shaped resonator, which exhibits a dipolar current distribution characterized by opposite current accumulation regions aligned along the incident electric-field direction. Finally, at 14.96 GHz, strong currents are simultaneously observed on both the central diamond resonator and the adjacent inner circular ring, revealing pronounced electromagnetic coupling and the formation of a hybrid resonant mode composed of coupled dipolar and loop-like current distributions. Overall, the surface current analysis confirms that the observed multiband absorption behavior originates from the progressive excitation of different resonator elements as the operating frequency increases. Furthermore, the current localization gradually shifts from the outer resonators toward the inner resonant structures, while the mutual electromagnetic coupling between adjacent elements contributes to the generation of additional absorption bands and enhanced resonance stability. Across all frequencies, the current distributions exhibit clear structural symmetry within each unit cell, confirming stable resonance behavior and polarization-insensitive absorption characteristics. Figure 10a–f also illustrate the electromagnetic field component magnitude variation of the proposed absorber at the same operating resonant bands, where the color scale from blue to red represents an increasing electric field. At 2.38 GHz, the Electromagnetic field component is strongly concentrated along the outer boundaries of the square unit cell and the outermost circular ring, with pronounced field enhancement near the square frame edges. This indicates dominant capacitive coupling between the incident wave and the outer resonator, corresponding to the lowest-frequency resonance. At 5.2 GHz, the high-intensity electric field shifts inward and becomes mainly localized on the outer circular ring, while the square boundary shows reduced excitation, confirming that the circular resonator governs this absorption band. At 6.8 GHz, the electric field is further confined toward the middle circular ring, forming a well-defined high-field region around its circumference, which demonstrates excitation of an intermediate electric resonance with limited involvement of the outer structures. At 9.7 GHz, the electric-field distribution becomes more compact and is primarily concentrated on the inner circular ring, with strong field accumulation visible along the upper and lower portions of the ring structure, indicating excitation of a higher-order localized electric mode. At 12.6 GHz, intense electric-field improvement is clearly identified around the central diamond-shaped resonator, while the surrounding circular rings exhibit comparatively weaker field intensities, confirming that the innermost metallic element dominates this resonance through strong capacitive effects. Finally, at 14.96 GHz, strong electric-field regions appear simultaneously around the central diamond and the adjacent inner circular ring, accompanied by noticeable field intensification at the inter-cell junctions, revealing strong electromagnetic coupling between closely spaced resonators at the highest operating frequency.

## 9. The Absorber Is Characterized by Its Frequency-Dependent Permittivity, Permeability, and Impedance

The behavior of the proposed metamaterial absorber (MMA) can be understood through its effective electromagnetic properties, specifically the relative permittivity ($\varepsilon_{r}$), relative permeability ($\mu_{r}$), and normalized impedance (*Z*). These quantities describe the interaction between the structure and the incident electromagnetic field and play a key role in determining its absorption performance. The effective parameters $\varepsilon_{r}$ and $\mu_{r}$ are retrieved from the simulated scattering data using the Nicolson–Ross–Weir (NRW) method, which utilizes the reflection ($S_{11}$) and transmission ($S_{21}$) coefficients. This approach is commonly employed to evaluate equivalent material properties from S-parameters. The corresponding relations are given by

(5) $$\varepsilon_{r}=\frac{c}{j2\pi ft}\cdot \frac{\left(1-S_{11}\right)-S_{21}}{\left(1+S_{11}\right)+S_{21}},$$

(6) $$\mu_{r}=\frac{c}{j2\pi ft}\cdot \frac{\left(1+S_{11}\right)-S_{21}}{\left(1-S_{11}\right)+S_{21}},$$

where *c* is the speed of light in free space, *f* denotes frequency, and *t* corresponds to the substrate thickness. Using these extracted parameters, the normalized impedance of the structure is obtained as

(7) $$Z=\sqrt{\frac{\mu_{r}}{\varepsilon_{r}}}.$$

**Figure 11:**
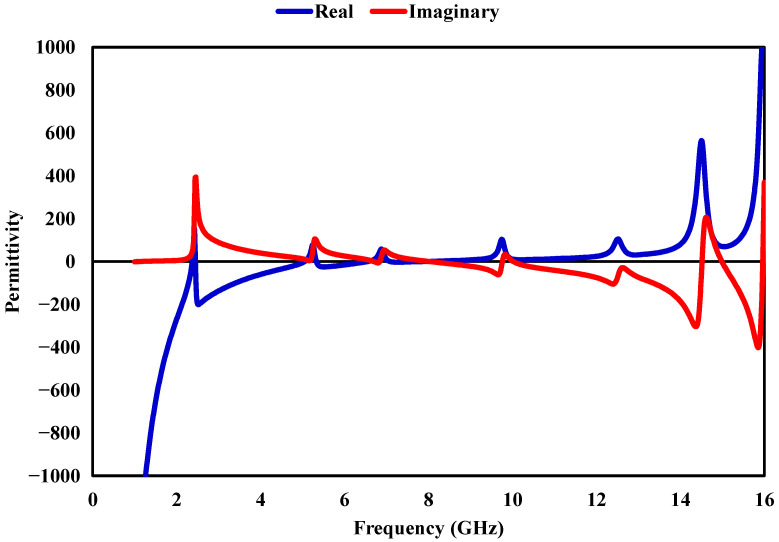
Real and imaginary components of the effective permittivity of the proposed MMA.

**Figure 12:**
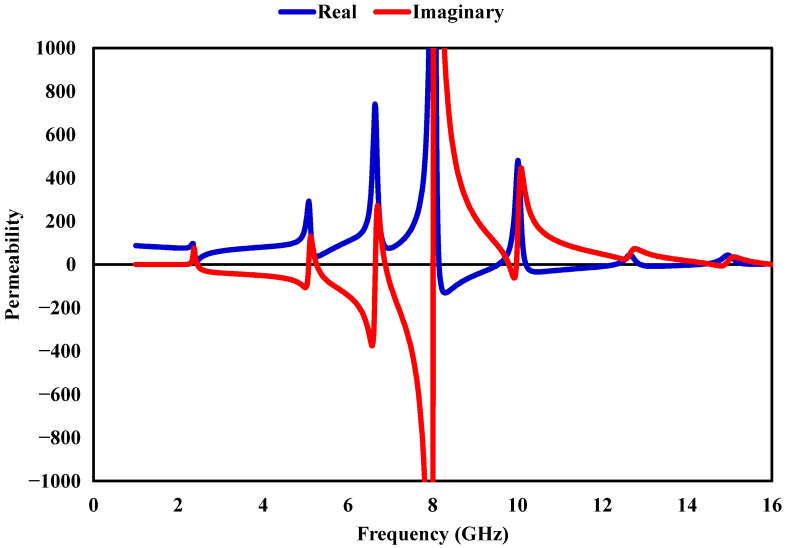
Real and imaginary components of the effective permeability of the proposed MMA.

**Figure 13:**
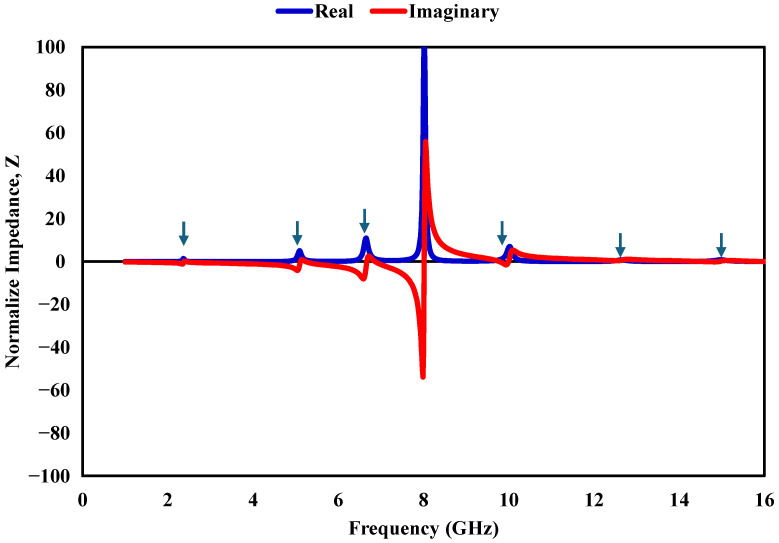
Normalized equivalent impedance response of the hexaband absorber.

Figure 11, Figure 12 and Figure 13 shows how $\varepsilon_{r}$, $\mu_{r}$, and *Z* vary with frequency. Near the resonances around 2.38, 5.2, 6.8, 9.7, 12.6, and 14.96 GHz, the real parts of $\varepsilon_{r}$ and $\mu_{r}$ exhibit opposite polarity, indicating strong resonant interaction within the absorber. At the same frequency, the imaginary components increase significantly, reflecting enhanced loss mechanisms that contribute to energy dissipation inside the structure. To further understand the absorption process, impedance matching is considered. When the input impedance approaches that of free space, reflection is minimized, which is a necessary condition for achieving high absorption. The reflection coefficient can be expressed as

(8) $$S_{11}=\left|\frac{Z/Z_{0}-1}{Z/Z_{0}+1}\right|,$$

where $Z_{0}=377\;\Omega$ represents the intrinsic impedance of free space. At approximately 2.38, 5.2, 6.8, 9.7, 12.6, and 14.96 GHz, the impedance approaches Z≈1+j0, indicating near-perfect matching with free space. Under this condition, reflection is significantly reduced, and the incident electromagnetic energy is effectively dissipated within the structure, leading to high absorption performance.

## 10. Measured Results

**Figure 14:**
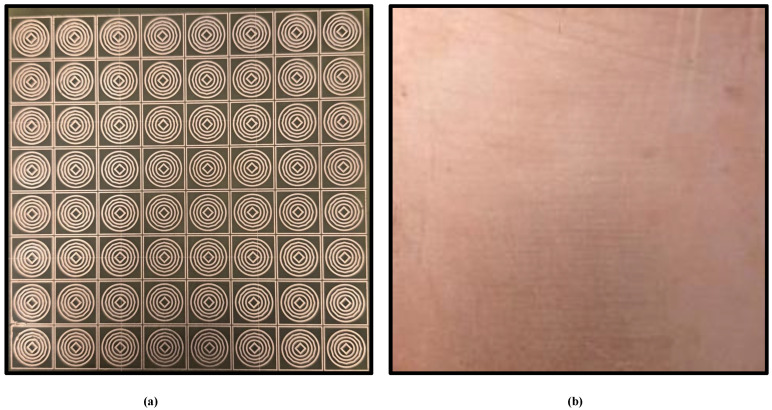
Fabrication and experimental validation of the proposed structure: (**a**) front view and (**b**) back view.

**Figure 15:**
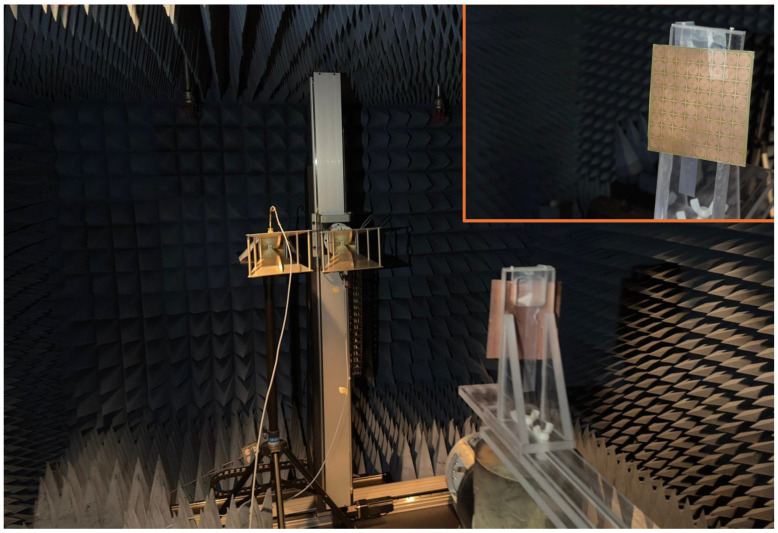
Block diagram representing the practical free-space measurement setup.

**Figure 16:**
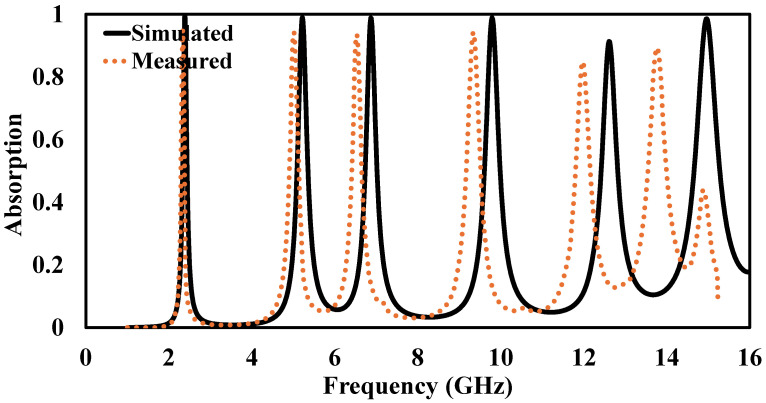
Comparison of simulated and measured absorptivity of the proposed metamaterial absorber.

To assess the practical performance of the proposed metamaterial absorber (MMA), a physical prototype was fabricated and experimentally characterized using a standard free-space reflection measurement setup. An image of the fabricated sample is presented in Figure 14. The prototype consists of an 8×8 periodic arrangement of unit cells, resulting in an overall size of $104\times 104\;{\mathrm{mm}}^{2}$, which is sufficiently large to approximate an effectively infinite surface under plane-wave illumination as shown in Figure 15. The experimental measurements were performed using an Agilent 8722ES vector network analyzer (VNA) connected to ultra-wideband horn antennas covering the wide operating frequency range. The transmitting and receiving horn antennas were positioned approximately 200 cm away from the fabricated sample to satisfy the far-field condition and ensure uniform plane-wave excitation across the absorber surface. Prior to the absorber measurements, a metallic copper plate with dimensions identical to the fabricated array was used as a reference reflector corresponding to nearly 100% reflection. In addition, a free-space measurement without the sample was conducted to account for environmental and system losses. The reflection coefficient of the proposed absorber was subsequently obtained through normalization with respect to the metallic reference plate. Since the proposed structure incorporates a continuous metallic ground plane, the transmission coefficient is zero. To investigate the finite-array effect, the fabricated prototype was implemented as a finite periodic array, whereas the numerical simulations were initially carried out under infinite periodic boundary conditions. The measured and simulated absorption responses shown in Figure 15 exhibit good overall agreement, thereby validating the proposed absorber design. The minor discrepancies observed, particularly within the 12–15 GHz frequency range, are mainly attributed to fabrication tolerances, finite-array truncation effects, slight misalignment during measurements, connector and cable losses, and small dimensional variations introduced during the fabrication process. Furthermore in Figure 16, the measured resonance frequencies are 2.40, 5.05, 6.55, 9.35, 11.95, and 13.75 GHz. The corresponding frequency shifts are 0.00%, 3.07%, 4.80%, 4.30%, 5.23%, and 8.27%, respectively. The absorption deviations are estimated to be 1.23%, 3.98%, 5.23%, 2.73%, 7.13%, and 9.43%, respectively. Overall, the experimental results confirm that the fabricated absorber maintains stable multiband absorption performance under practical measurement conditions, demonstrating its suitability for real-world electromagnetic and microwave applications.

## 11. Comparative Study with Previously Published Metamaterial Absorbers

Table 2 presents a comparative evaluation of the proposed metamaterial absorber with previously reported designs in terms of unit-cell size, operating frequency bands, absorption efficiency, polarization insensitivity, and application areas. It can be observed that earlier works demonstrate high absorption performance; however, many are limited either in terms of operating frequency range, polarization dependence, or application diversity. In contrast, the proposed design exhibits a compact unit-cell size of 13×13 mm^2^ while supporting multiple resonant frequencies spanning from 2.4 GHz to 15 GHz. Compared to existing studies, which typically operate over fewer bands or narrower frequency ranges, the proposed absorber achieves a wider multi-band response. Additionally, the absorption levels consistently remain above 97%, indicating superior efficiency across all operating bands. Furthermore, while some reported designs lack polarization-insensitive behavior, the proposed absorber maintains stable performance under different polarization conditions, enhancing its practical applicability. The ability to operate across S-, C-, X-, and Ku-bands further distinguishes the proposed structure from prior works, which are often limited to specific applications such as WLAN, radar, or energy harvesting. Overall, the comparison demonstrates that the proposed MMA offers a favorable balance between compact size, wide frequency coverage, high absorption efficiency, and polarization-independent operation, making it a competitive candidate for advanced electromagnetic and microwave applications.

## 12. Conclusions

This work focuses on the design, numerical analysis, and validation of a multi-band metamaterial absorber based on a coupled resonator architecture, achieving near-unity absorption at distinct microwave frequencies. The absorber geometry is realized through a systematic design evolution that begins with a fundamental square resonator and progressively incorporates circular, dodecagonal, and hybrid resonant elements. This structured development enhances electromagnetic coupling and enables the excitation of multiple resonant modes, leading to six well-defined absorption bands centered at 2.4 GHz, 5.21 GHz, 6.88 GHz, 9.77 GHz, 12.61 GHz, and 14.99 GHz. Full-wave electromagnetic simulations confirm that absorption efficiencies exceed 97% at most resonance frequencies, with peak absorptivity approaching unity. The absorber also exhibits polarization-insensitive behavior and maintains stable performance under wide-angle oblique incidence, demonstrating robustness under practical excitation conditions and suitability for multiband microwave applications. To elucidate the absorption mechanism, the contributions of individual resonant elements are systematically examined. The results indicate that the high absorption originates from the combined effects of electric dipole resonances and inter-element coupling. Further analysis of the electric field distributions, surface current distributions, and normalized surface impedance confirms that effective impedance matching between the metasurface and free space, together with intrinsic resonant losses, governs the absorption behavior. The proposed hexaband absorber provides a scalable and versatile platform for multiband absorption engineering. Its compact structure, high efficiency, and stable angular and polarization response allow straightforward extension toward additional functionalities, such as mechanical flexibility or optical transparency. As a result, the design shows strong potential for advanced electromagnetic applications, including electromagnetic interference mitigation, stealth technologies, and multiband microwave systems. Overall, this study offers both fundamental insight and practical guidance for the development of high-performance metamaterial absorbers. 

## Figures and Tables

**Table 1:** Optimized design variables of the Developed Model Metamaterial Absorber.

Design Variables	Size (mm)	Design Variables	Size (mm)
$w_{s}$	13	$l_{s}$	13
$w_{g}$	13	$l_{g}$	13
$w_{p}$	12.75	$l_{p}$	12.75
*R*	5.5	$R_{1}$	4.5
$R_{2}$	3.5	$R_{3}$	2.5
$s_{q}$	2.5	$h_{c}$	0.035

**Table 2:** Comparative study between the proposed metamaterial absorber and previously reported designs.

Ref.	Year	UC Size	Substrate Thickness (mm)	Freq. (GHz)	Abs. (%)	PI	Application
[42]	2023	20×20	1.6	2.4, 3.5, 5.8	99.3, 95.6, 99.5	Yes	WLAN, 5G
[43]	2019	10×10	1.6	5.57, 7.97, 13.44	98.9, 97.9, 99.28	Yes	Radar detection
[44]	2019	24×24	1.6	8.6, 10.2, 11.95	>80	Yes	X/Ku
[45]	2022	28.3×28.3	1.5	2.4, 5.2, 5.8	99.98	No	Energy harvesting
[46]	2023	17×17	1.6	20.38–25.12	97.8–99.3	Yes	5G mm waves applications
[47]	2021	10.4×10.4	1.6	3.2, 5.32, 11.15, 16.73	95.7–97.7	Yes	S/C/X bands
[48]	2022	8×8	0.8	24, 28	98, 94	No	5G mm waves applications
[49]	2025	24×24	1.6	1.8, 3.5	98.7, 99.7	Yes	EMI shielding
[50]	2025	15.7×15.7	1	2.8, 6.32, 9.17, 12.21, 14.4, 17.3, 19.5	92.6, 91.7, 93, 96.3, 97.4, 98.3, 99.8	Yes	S/C/X/Ku/K bands
[51]	2026	12.5×12.5	1.6	2.178, 5.484, 8.391, 11.811, 15.858, 18.689	99, 98, 99, 99, 99, 97	Yes	S/C/X/Ku bands
[52]	2026	10×10	1	5.4, 9.65, 15.73	96, 99, 98	Yes	C/X/Ku bands
**Prop.**	**2026**	13×13	1.6	**2.4, 5.21, 6.88, 9.77, 12.61, 15**	**98.23, 97.98, 98.23, 97.73, 91.13, 97.43**	**Yes**	**S/C/X/Ku bands**

## Data Availability

The original contributions presented in this study are included in the article. Further inquiries can be directed to the corresponding author.
